# Allometric relationships between stem diameter, height and crown area of associated trees of cocoa agroforests of Ghana

**DOI:** 10.1038/s41598-023-42219-6

**Published:** 2023-09-09

**Authors:** Michael Asigbaase, Evans Dawoe, Simon Abugre, Boateng Kyereh, Collins Ayine Nsor

**Affiliations:** 1 Department of Forest Science, School of Natural Resources, University of Energy and Natural Resources, Sunyani, Ghana; 2 Department of Agroforestry, Faculty of Renewable Natural Resources, Kwame Nkrumah University of Science and Technology, Kumasi, Ghana; 3 Department of Silviculture and Forest Management, Faculty of Renewable Natural Resources, Kwame Nkrumah University of Science and Technology, Kumasi, Ghana; 4 Department of Forest Resources Technology, Faculty of Renewable Natural Resources, Kwame Nkrumah University of Science and Technology, Kumasi, Ghana

**Keywords:** Ecology, Agroecology, Ecosystem ecology, Tropical ecology, Ecology, Agroecology, Ecosystem ecology, Tropical ecology

## Abstract

Allometric models which are used to describe the structure of trees in agroforestry systems are usually extrapolated from models developed for trees in forest ecosystems. This makes quantitative assessment of the functions of shade trees in agroforestry systems challenging since increased availability of light and space in these systems may induce structural differences from those growing under forest conditions. We addressed this issue by providing species-specific allometric information on the structural characteristics of associated shade trees on cocoa agroforestry systems and assessed if allometries conformed to theoretical predictions. At the plot level, stand and soil characteristics affecting tree structural characteristics were assessed. The study was conducted in cocoa agroforestry systems at Suhum, Ghana. The height-diameter at breast height (H-DBH) allometry had the best fits (R^2^ = 53–89%), followed by the crown area (CA)-DBH allometry (R^2^ = 27–87%) and then the CA-H allometry (R^2^ = 22–73%). In general, the scaling exponents of the CA-DBH, H-CA and H-DBH allometries conformed to the metabolic scaling theory (MST). However, both the CA-DBH and H-DBH allometries diverged from the geometric similarity model. Though forest tree species had similar crown areas as fruit trees, they were slenderer than fruit trees. Tree slenderness coefficients were positively correlated with soil P, Ca, Cu and the ratios (Ca + Mg):K, (Ca + Mg):(K + Na) and Ca:Mg, but not C:N while DBH and H were correlated with soil P and C:N ratio. Our results show that critical soil nutrients and their ratios affects shade tree structural attributes (e.g. slenderness and CA), which possibly restrict variations in species-specific allometries to a narrow range on cocoa systems. Furthermore, shade tree species richness and density are better predictors of relative canopy projection area (a proxy for shade intensity) than tree species diversity. In conclusion, the results have implications for shade tree species selection, monitoring of woody biomass and maintenance of biodiversity.

## Introduction

Cocoa agroforestry systems are complex dynamic systems whose structure and composition depend on the management approach farmers adopt and their preferences in relation to which shade trees (i.e. trees retained or planted on cocoa farms to provide shade for cocoa trees and other non-cocoa products such as fruits or timber) to maintain on their farms^1,2^. However, most allometric equations used to describe the structure of trees in agroforestry systems are extrapolated from allometric equations developed for trees in forest ecosystems. For example, widely used pan-tropical generalized allometric equations for biomass and carbon estimation in forest systems [e.g., Refs.^3–6^] are generally applied in agroforestry ecosystems [e.g., Refs.^7–11^]. While generic equations provide a convenient approach for estimating tree attributes across a broad range of species, recent advances in ecological science emphasize the significance of species-specific equations. These equations offer improved accuracy, context-specific estimations, and enhanced precision, making them essential tools in contemporary ecological studies. As ecological research continues to evolve, the importance of species-specific allometries in understanding tree growth and attributes cannot be overstated. For example, Chave et al.’s^12^ models overestimated aboveground biomass by 40% in Central Africa^13^ due to differences in height and crown width allometries between trees in Central Africa and those within the geographic range where the models were developed^14–16^. In Africa, a few species-specific equations are available, but they generally do not focus on the height-crown area or diameter-crown area allometries [e.g. Refs.^1,11^]. Furthermore, it is laborious, expensive and difficult to measure tree crowns and heights in both cocoa agroforestry and forest ecosystems. Thus, diameter at breast height still remains a quick and easy-to-measure proxy for the estimation of other dimensions, such as tree height and crown variables (e.g., crown area, diameter, radius, and cover) in both agroforestry and forest ecosystems^10,17,18^. However, applying tree allometric equations developed for forest ecosystems in cocoa agroforestry systems may lead to errors because allocation of resources may change in response to increased space and light availability in cocoa agroforestry systems^19,20^.

The allocation of resources in trees and consequently their influence on tree allometries is primarily dependent on availability of space and access to light^19–21^. Increase in light availability and lateral empty space in cocoa agroforestry systems might promote horizontal crown expansion, thereby increasing the amount of carbon stored in tree branches^20,21^. Furthermore, crown expansion results in the thickening of the tree stem base to provide mechanical support at the expense of vertical growth which could potentially influence tree allometries^19,21^. Tree diversity, soil characteristics, the so-called home advantage or local environment of an individual tree and species-specific traits have been shown to affect tree allometries^10,19,20,22–24^. Therefore, tree allometries which reflect agroforestry ecosystems are urgently needed for estimation of components that deepen our understanding of these systems.

Tree allometries of cocoa agroforestry systems may scale differently from those growing in forest ecosystems due to differences in species composition and the micro-environment. Biophysical models (e.g. metabolic scaling theory, MST) and physical ones (geometric, elastic and stress similarity) have been proposed to describe tree allometries, but it is unclear whether associated trees on cocoa farms conform to the predictions of these theories given their peculiar species composition and environment. The geometric similarity is based on isometrical scaling of organ length and radius and predicts a direct proportional scaling between diameter at breast height (DBH) and tree height^25^. The elastic similarity is based on margin safety of branches against mechanical failure^26^, while the constant stress similarity model, which is based on the stress produced by wind pressure along the stem, predicts stem diameter to scale as the 2nd power of height^27^. On the other hand, MST is based on the maximization of metabolic rate and predicts stem diameter to scale as 3/2 power of height^28^. While the predictions of these theories have been tested in relation to the CA-DBH and DBH-H allometries, the results have been non-conclusive^10,19,21^. Furthermore, to the best of our knowledge, the predictions of these theories have not been extended to the CA-H allometry.

That notwithstanding, tree slenderness coefficient, defined as the ratio of the total height (H) to diameter at breast height (1.3 m above the ground, DBH) when both H and DBH are measured in the same units (e.g., metres), is an index of tree stability or the resistance to windthrow. The larger the slenderness coefficient of a tree, the higher its susceptibility to wind damage and vice versa. Tree attributes (e.g., DBH, height, crown shape and size, and root system) and site condition, including soil characteristics, and their interaction strongly influence tree slenderness coefficients^29^. Tree stability or their resistance to winds may affect tree allometries. How tree slenderness which shapes the scaling exponents of trees in agroforestry systems is affected by stand and soil characteristics remains yet to be understood.

Tree allometries play a critical role in the accurate assessment of tree and agroforestry biomass from local to global scales. It also essential in linking datasets acquired by air and space borne sensors to ground-based datasets for large scale assessments of agroforestry systems. Our major research questions were: (i) does the scaling exponents of DBH, H and CA allometries conform to predictions of biophysical (e.g. metabolic scaling theory, MST) and physical (e.g. geometric, elastic and stress similarity) models? (ii) how does stand and soil characteristics affect tree slenderness? (iii) what is the relationship between relative sum of crown projection area (RSCA) and stand characteristics? Consequently, the objectives of the study were: (i) to provide species-specific allometries of frequently used shade tree species in cocoa agroforestry systems and assess if allometries conformed to the physical models (geometric and stress similarity) or biophysical ones (metabolic scaling theory, MST); (ii) to evaluate how tree slenderness (a proxy for tree resistance to windthrow) is affected by stand and soil characteristics; (iii) to determine the relationship between tree slenderness and crown area (CA). At the plot level, (iv) we assessed whether soil and stand characteristics affect mean DBH, height, crown area and wood density and (v) determined the relationship between relative sum of crown projection area (RSCA) and stand characteristics. RSCA is the sum of the crown projection area of a stand divided by the stand area multiplied by 100. RSCA is an indication of shade intensity and a proxy for both leaf area and light interception. For example, a RSCA of more than 100 means some parts of the stand have multi-coverage.

## Methods

### Study sites

**Figure 1:**
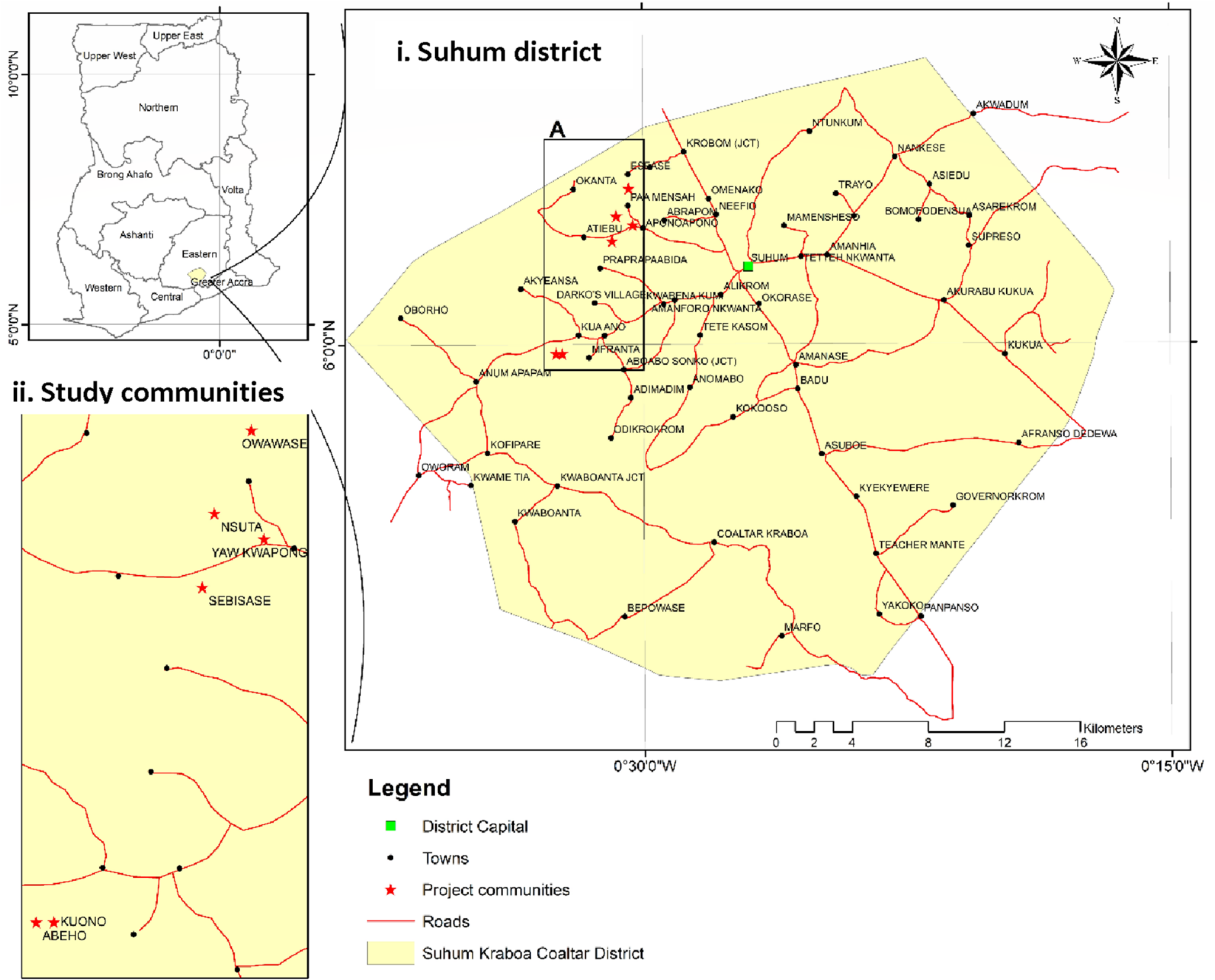
Map of study area; showing Suhum district (panel i) and the study communities (panel ii) (Authors construct, ArcGIS version 10.4.1, https://desktop.arcgis.com/).

Our study datasets were collected from Suhum (N 06° 5′ and W 0° 27′) in the Eastern Region of Ghana (Fig. 1). The study area falls within the Moist Semi-deciduous Forest Zones with two wet seasons (major season; April to July and minor season; September to November) and two dry seasons (major season; December to March and minor season; August). Rainfall ranges from 1270 to 1651 mm per annum and average ambient temperatures are uniformly high throughout the year and range from 24 to 29 °C. The cocoa stands in the study districts were agroforestry systems with variable proportions of naturally regenerated or planted forest tree species, fruit trees and food crops. The predominant forest species included *Terminalia ivorensis* (A. Chev.), *Entandophragma angolense* (Welw.) C.DC, *Morinda lucida* Benth., *Holarrhena floribunda* (G. Don) Dur and Schinz, and *Spathodea campanulata* P. Beauv.The fruit trees were *Citrus sinensis* (L.) Osbeck, *Persea americana* Mill. and *Mangifera indica* L. Soils in the study area are classified as forest ochrosols^30^.

### Field data attributes

Data sets were collected in 84 plots of dimensions 25 m × 25 m (established by 2). In brief, the plots were established by adopting a multi-stage approach in the selection of study communities and cocoa farms/farmers. First, Suhum Municipality was purposely selected because cocoa production under shade trees is encouraged. Second, seven communities known to produce cocoa under shade trees were randomly selected from a list provided by the Municipal office of the Ghana COCOBOD (the regular of the cocoa sector). Finally, 84 farms/farmers were randomly selected from a list of farmers in the selected communities provided by the regulators. The age of the selected cocoa farms ranged from 4 to over 50 years. The circumference of all trees greater than 15 cm at 1.3 m above the ground (i.e. DBH > 5 cm) were measured using tape measures (in cm) and later converted to diameter values. A total of 551 individual trees belonging to 82 species and 26 families were inventoried. Shade trees on cocoa farms were formally identified (botanical and local names) in the field by an experienced forest taxonomist and technician (Mr. Jonathan Darbo) from the Council for Scientific and Industrial Research and two key informants based on Hawthorne and Jongkind^31^. No plant voucher specimens were collected as all plants all identified in the field. Tree heights (distance from the ground to the highest point of the tree crown) were measured using Vertex IV and Transponder (Haglöf Sweden). Crown length (in m) was measured along the largest crown diameter from one end of the tip of the crown base to the other. Crown width (in m) was measured along the diameter perpendicular to the crown length. The total crown cover in all data sets were expressed as a percentage per ha. Crown areas (CA) were estimated assuming a circular crown, with a radius estimated as half of the average of all diameters, including the largest diameter. Supplementary Tables 1 and 2 in Supplementary Material 1 (online) provide a summary of the structural attributes of the datasets of all trees in the cocoa agroforestry systems as well as the soil nutrient stocks. Data on stand characteristics (such as species richness, Shannon diversity and standing litter stock) and soil nutrient stocks were obtained from Asigbaase et al.^2,32,33^; details on data collection methods are provided in Supplementary Information 1 in Supplementary Material 1 (online). Our study complies with relevant institutional, national, and international guidelines and legislation on plants identification in the field. Our study was approved by the Department of Forest Ethics Committee, University of Energy and Natural Resources, Ghana and the School Research Ethics Committee of the University of Nottingham, UK. Cocoa farmers/landowners provided written informed content (or informed oral consent) to the data collection and we obtained permission (written content) from the Ghana Cocoa Board (COCOBOD), a government institution which is the sole regulator of the cocoa production sector in the country.

### Data analysis

The dataset of all plots/farms were partitioned into within cocoa canopy shade trees (which corresponds to trees with relatively small diameter) and above cocoa canopy shade trees (which corresponds to emergent trees with large diameter) based on the mean height of cocoa trees in each plot/farm^34,35^. This accounted for the variability in tree structural characteristics that may exist between different strata of the cocoa ecosystem. The differences in tree structural characteristics between forest and fruit shade trees within and above the cocoa canopy were assessed using Kruskal–Walli’s rank sum test.

Species-specific allometries were fitted for tree species represented by at least ten individuals Feldpausch et al.^19^. Log–log regression has proven to provide satisfactory allometries for tropical trees around the world and for forest inventory data sets, including Ghana [e.g., Refs.^19,36–39^]. It acceptably rendered the allometries with lower AIC and BIC and higher adjusted R^2^ values compared to other models (quadratic, Chapman-Richard, exponential, Gompertz, hyperbolic, logistic, Michaelis–Menten (saturation growth), power-law, Richard and Weibull functions), and was thus used to fit all allometric relationships. Specifically, we used the function: y = m × z^n^, linearized as log (y) = log m + n × log z. In fitting the tree allometries, y was alternatively CA or H and z was alternatively DBH or H. The intercept and scaling exponent in the function are m and n, respectively. The intercept (m) is the ratio of specific constraints for y to specific constraints for z^39^. That means larger m values will result in smaller values for y. The relationship between the relative sum of canopy projection area (RSCA) and tree density, species richness and Shannon diversity were determined via the via regression analysis, with RSCA as dependent variable and tree density, species richness and Shannon diversity as independent variables. The back-transformation of the log–log linear values into the original scale may induce bias. Thus, to remove bias, the predictions were adjusted using the smearing method (i.e. predicted values were multiplied by expo(0.5 × s^2^), where s is the variance)^39,40^.

The slenderness coefficient for an individual tree was estimated as the ratio of its total height (m) to its DBH (m). The relationship between tree CA and slenderness coefficient for each species with individuals ≥ 10 was assessed via regression. One-way ANOVA was used to determine whether differences in slenderness coefficients were significant among the shade tree species; this was followed by Tukey–Kramer post hoc test to establish difference between pairs of means. Furthermore, tree dimensions (slenderness coefficient, DBH, H, mean wood density, and total CA) were related to soil nutrient stocks and stand parameters (farm size, species richness, Shannon diversity, stand basal area, litter stock, and tree density) via Spearman’s rank correlation to assess how environmental factors affected tree parameters.

The confidence interval of the scaling exponents of species-specific allometries were examined to determine they bracketed the predictions of the four biophysical and physical models (Table 1)^35,41,42^. In addition, one-sample t-test was conducted to assess whether the scaling exponents of tree allometries conformed to the predictions of models evaluated in the study (Table 1). Based on the predictions of the four models tested in this study and assuming isometric scaling between (i) crown radius and DBH and (ii) crown radius and H, crown area will scale in unity to height (stress similarity model) and to the second power of height (geometric similarity model)^25–27,43^. Given that the MST predicts (i) height to scale to a 2/3 power of diameter and (ii) diameter to scale to a 4/3 power of CA (28), then CA will scale to the second power of H. The normality of the residuals of the datasets after fitting the allometries was tested using Shapiro-Wilks W-test for homogeneity of variances. Where applicable in the analysis, whenever the requirements for parametric tests (e.g. ANOVA) were not met, non-parametric approaches (e.g. Kruskal–Wallis rank test) were used. An alpha (α) value of 0.05 was applied in all the analysis.

**Table 1:** Theoretical predictions of tree allometric exponents.

Theory predictions	H-DBH	CA-DBH	CA-H
Stress similarity	½	–	(1)
Geometric similarity	1	(2)	–
Elastic similarity	2/3	–	–
MST	2/3	4/3	(2)

Predicted allometric exponents in parenthesis are proposed in this study based on the predictions for the H-DBH and CA-DBH allometries by the respective models and assuming isometric scaling. Unbracketed predicted allometric exponents were taken from McMahon^26^, Dean and Long^27^, Norberg^25^, West et al.^28^, Sun et al.^44^ and Shenkin et al.^38^.

## Results

### Overview of dimensions of shade trees on cocoa farms

Forest trees within the cocoa canopy layer were slender with lower wood density than fruit trees (Table 2). However, median DBH, basal area, and crown area were similar. The same pattern was observed for shade trees above the cocoa canopy layer.

**Table 2:** Structural characteristics of forest and fruit shade tree species within and above the cocoa tree canopy at Suhum.

Parameter	Category	N	Median	H	*p*
Within cocoa canopy trees					
DBH cm	Forest tree	249	15.27	3.23	0.072
	Fruit tree	63	17.56		
Basal area	Forest tree	249	0.01833	3.23	0.072
	Fruit tree	63	0.02424		
Wood density	Forest tree	249	0.51	43.09	< 0.001
	Fruit tree	63	0.62		
Slenderness coefficient	Forest tree	249	74.1	11.42	0.001
	Fruit tree	63	58.1		
Crown area	Forest tree	249	22.91	0.47	0.491
	Fruit tree	63	21.66		
Above cocoa canopy trees					
DBH cm	Forest tree	213	26.41	0.02	0.89
	Fruit tree	26	25.68		
Basal area	Forest tree	213	0.0548	0.02	0.89
	Fruit tree	26	0.05185		
Wood density	Forest tree	213	0.51	12.87	< 0.001
	Fruit tree	26	0.56		
Slenderness coefficient	Forest tree	213	72.96	11	0.001
	Fruit tree	26	54.06		
Crown area	Forest tree	213	41.02	1.44	0.231
	Fruit tree	26	48.74		

### Species-specific allometries of shade trees on cocoa farms

All species-specific allometric relationships, i.e., H-DBH, CA-DBH and CA-H, followed a log-linear model (Table 3), with better fits for the H-DBH allometry (R^2^ = 53–89%), followed by the CA-DBH allometry (R^2^ = 27–87%) and then the CA-H allometry (R^2^ = 22–73%). The scaling exponents of the H-DBH relationship were smaller and narrower in range compared to the CA-DBH and CA-H relationships (*p* < 0.001); Tukey–Kramer post-hoc analysis showed that the mean scaling exponents of the allometries decreased in the order CA-H > CA-DBH > H-DBH (Table 4). In contrast, the intercepts of the CA-H, H-DBH and CA-DBH allometries were similar.

**Table 3:** Relationship between crown area, DBH and height of commonly used shade trees in cocoa growing systems at Suhum and their conformity to predicted allometric exponents of four biophysical models.

Allometry	Species	No	Slope	Intercept	R^2^ (%)	SE	*p*	*MST*	*GS*	*ES*	*SS*
CA-DBH	*C. sinesis*	36	1.30 (0.96, 1.64)	2.71 (2.41, 3.51)	62.9	0.210	***	✓	x	–	–
	*E. angolense*	17	1.12 (0.70, 1.53)	2.16 (1.61, 4.08)	66.6	0.198	***	✓	x	–	–
	*F. sur*	14	0.97 (0.35, 1.58)	4.28 (2.36, 2.36)	45	0.321	**	✓	x	–	–
	*H. floribunda*	42	1.42 (1.06, 1.78)	2.95 (2.73, 3.60)	60.8	0.213	***	✓	x	–	–
	*M. indica*	12	1.74 (1.27, 2.21)	1.27 (1.11, 2.18)	87.4	0.114	***	✓	✓	–	–
	*M. regia*	13	0.72 (0.04, 1.40)	6.34 (2.39, 36.55)	26.9	0.300	*	✓	x	–	–
	*M. zechiana*	13	1.57 (1.01, 2.12)	1.60 (1.31, 2.65)	76	0.169	***	✓	✓	–	–
	*N. laevis*	17	1.49 (0.78, 2.20)	2.93 (2.77, 4.09)	55.9	0.344	***	✓	✓	–	–
	*R. vomitoria*	15	0.78 (0.11, 1.45)	5.08 (2.48, 21.03)	27.7	0.302	*	✓	x	–	–
	*S. campanulata*	11	0.85 (0.32, 1.38)	3.47 (1.68, 14.09)	55	0.225	**	✓	x	–	–
	*T. ivorensis*	23	1.16 (0.79, 1.53)	3.46 (2.35, 7.15)	67	0.233	***	✓	x	–	–
H-DBH	*C. sinesis*	39	0.42 (0.29, 0.54)	4.24 (3.31, 5.57)	53.4	0.077	***	x	x	x	✓
	*E. angolense*	17	1.00 (0.69, 1.30)	1.95 (1.50, 3.08)	75	0.145	***	✓	✓	✓	x
	*F. sur*	14	0.50 (0.30, 0.71)	4.40 (2.80, 7.48)	67.8	0.106	***	✓	x	✓	✓
	*H. floribunda*	47	0.53 (0.39, 0.67)	4.90 (3.67, 6.76)	55.4	0.091	***	✓	x	✓	✓
	*M. indica*	12	0.52 (0.26, 0.77)	3.32 (1.90, 7.01)	64.1	0.063	**	✓	x	✓	✓
	*M. regia*	12	0.46 (0.26, 0.66)	4.49 (2.91, 7.41)	70	0.081	***	✓	x	✓	✓
	*M. zechiana*	13	0.73 (0.48, 0.99)	2.81 (2.02, 4.23)	76.4	0.078	***	✓	x	✓	✓
	*N. laevis*	17	0.71 (0.43, 0.99)	3.21 (2.13, 5.50)	63.3	0.139	***	✓	x	✓	✓
	*R. vomitoria*	15	0.57 (0.42, 0.73)	3.16 (2.42, 4.30)	81.2	0.071	***	✓	x	✓	✓
	*S. campanulata*	10	0.65 (0.47, 0.82)	3.13 (2.17, 4.92)	88.6	0.069	***	✓	x	✓	✓
	*T. ivorensis*	23	0.54 (0.40, 0.69)	4.35 (3.13, 6.32)	73	0.093	***	✓	x	✓	✓
CA-H	*C. sinesis*	33	2.03 (0.90, 3.17)	4.65 (4.49, 6.87)	27.8	0.301	**	✓	✓	–	–
	*E. angolense*	17	2.07 (1.36, 2.78)	1.30 (1.25, 1.71)	73.4	0.159	***	✓	x	–	–
	*F. sur*	14	1.81 (0.90, 2.71)	2.04 (1.76, 5.62)	58	0.280	***	✓	✓	–	–
	*H. floribunda*	42	1.34 (0.64, 2.03)	5.53 (5.31, 7.15)	31.9	0.282	***	✓	✓	–	–
	*M. indica*	12	2.35 (1.04, 3.66)	1.46 (1.28, 8.51)	60.6	0.201	**	✓	x	–	–
	*M. regia*	12	1.94 (0.88, 3.01)	1.65 (1.44, 5.11)	58.4	0.234	**	✓	✓	–	–
	*M. zechiana*	13	1.75 (0.92, 2.58)	1.61 (1.37, 3.20)	63.2	0.209	***	✓	✓	–	–
	*N. laevis*	17	1.63 (0.73, 2.53)	3.43 (3.28, 4.93)	48.3	0.373	**	✓	✓	–	–
	*R. vomitoria*	15	1.12 (0.02, 2.23)	4.01 (2.25, 26.47)	21.5	0.314	*	✓	✓	–	–
	*S. campanulata*	10	1.43 (0.61, 2.24)	1.83 (1.34, 7.45)	62.9	0.215	**	✓	✓	–	–
	*T. ivorensis*	23	1.57 (0.82, 2.32)	3.46 (2.85, 8.97)	46.2	0.298	***	✓	✓	–	–

*MST* metabolic scaling theory, *GS* geometric similarity theory, *ES* elastic similarity theory, *SS* stress similarity theory, *SE* standard deviation, *No.* number of trees. The 95% confidence interval of the slope and intercepts are in parenthesis. *p < 0.05; **p < 0.01; ***p < 0.001.

**Table 4:** Comparison of all trees allometries, including testing the predicted allometric exponents of four biophysical models, using log-transformed data.

Allometry	Slope	SD	Intercept	SD	Theory prediction			
					MST	GST	EST	SST
CA-DBH	1.192 (1.006, 1.377)^b^	0.34	3.295 (2.486, 4.105)^a^	1.493	4/3	2	–	–
H-DBH	0.602 (0.417, 0.787)^c^	0.163	3.633 (2.824, 4.443)^a^	0.9	2/3	1	2/3	1/2
CA-H	1.730 (1.545, 1.915)^a^	0.36	2.817 (2.007, 3.626)^a^	1.465	2	–	–	1

*SD* standard deviation, *MST* metabolic scaling theory, *GST* geometric similarity theory, *EST* elastic similarity theory, *SST* stress similarity theory. Mean slope and intercepts values (n = 11) are shown with 95% confidence interval in parenthesis. The theory prediction is underlined if it is similar to the slope (*t-test*) and falls within the 95% confidence interval of the slope. The letters (a, b, c) indicate the results of the Tukey–Kramer post-hoc test between the slopes and the intercepts.

**Figure 2:**
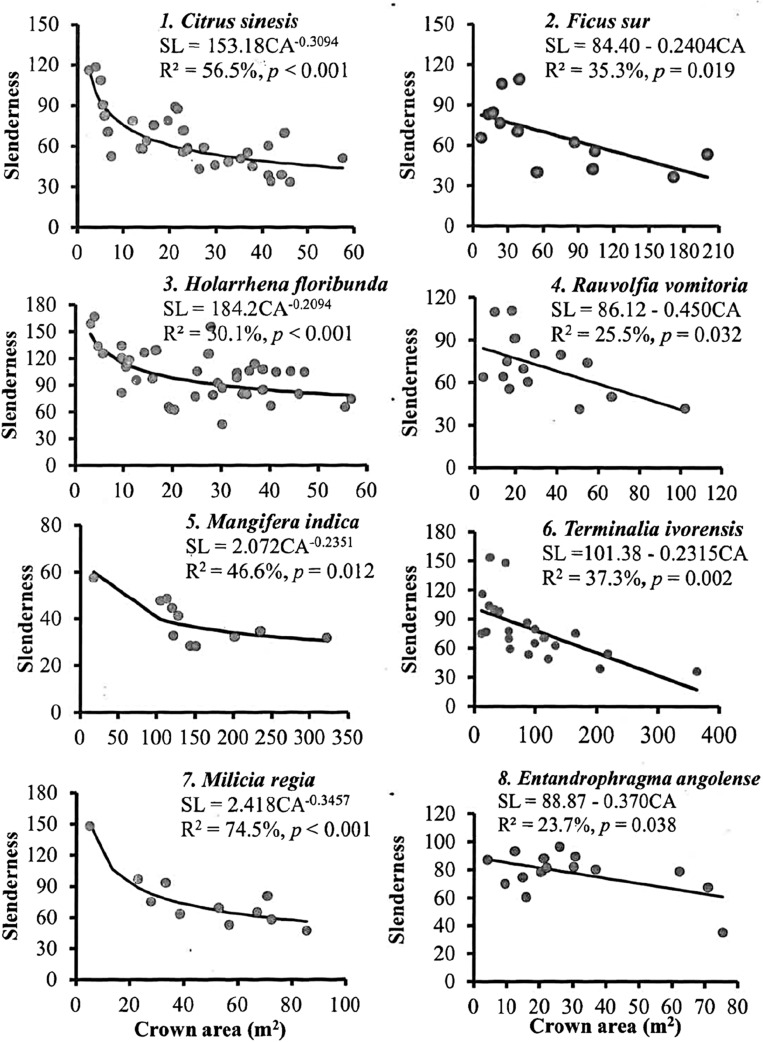
Relationship between slenderness coefficients and crown area of frequently used shade trees on cocoa farms at Suhum.

In general, the mean scaling exponents of the CA-DBH allometry conformed to the MST (n = 1.3; t-test, *p* = 0.316), but violated the geometric similarity model (n = 2; t-test, *p* < 0.001) predictions (Table 4). The H-DBH allometry conformed to the MST (*p* = 0.215), elastic similarity (*p* = 0.215) and stress similarity predictions (*p* = 0.064), but had significantly lower scaling exponents compared to those predicted by the geometric similarity model (*p* < 0.001). In general, the CA-H allometry exponent conformed to the prediction of the stress similarity model and the MST. Slenderness coefficients differed significantly among the frequently used shade tree species (*p* < 0.001); Table 5 provide details on Tukey–Kramer pairwise comparisons for the evaluated species. The relationship between CA and slenderness coefficients were significant for all species (R^2^ = 23.6–75.9%), except *Millettia zechiana, Spathodea campanulata* and *Newbouldia laevis* (Fig. 2).

**Table 5:** Mean slenderness coefficient (SL) of frequently used shade trees on cocoa farms at Suhum. *No.*number of individuals, *SD* standard deviation. The letters (a, b, c, d) indicate the results of Tukey–Kramer pairwise post-hoc comparison.

Species	No	Mean SL (95% CI)	SD
*C. sinesis*	39	65.38 (56.73, 74.03)^c,d^	21.68
*E. angolense*	16	80.15 (66.65, 93.66)^a,b,c^	17.71
*F. sur*	14	75.81 (61.38, 90.25)^a,b,c^	36.51
*H. floribunda*	47	101.02 (93.14, 108.90)^a^	28.12
*M. indica*	12	38.76 (23.17, 54.36)^d^	9.21
*M. regia*	13	68.06 (53.08, 83.04)^b,c,d^	34.95
*M. zechiana*	13	97.37 (82.39, 112.35)^a,b^	18.32
*N. laevis*	17	99.9 (86.8, 113.0)^a,b^	41.70
*R. vomitoria*	15	71.32 (57.37, 85.27)^b,c,d^	21.24
*S. campanulata*	11	59.93 (43.64, 76.21)^c,d^	26.61
*T. ivorensis*	23	79.38 (68.12, 90.65)^a,bc^	30.03

**Figure 3:**
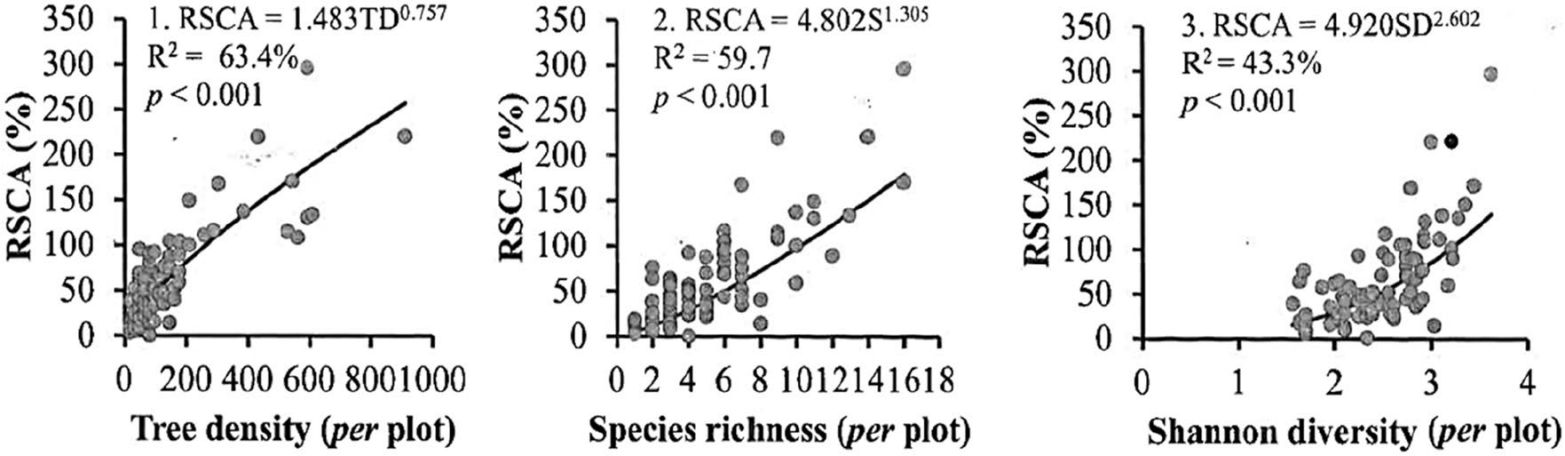
Relationship between relative sum of canopy projection area (RSCA) and tree density, species richness and Shannon diversity in cocoa agroforestry systems at Suhum.

At the plot level, relative sum of crown projection area (RSCA)-species richness, RSCA-species diversity, and RSCA-tree density allometries followed a log-linear model (Fig. 3). Tree density (R^2^ = 63%) and species richness (R^2^ = 60%) explained RSCA better than species diversity (R^2^ = 43%). Both mean DBH and mean H were not related to RSCA.

### Soil factors affecting tree parameters

We assessed the relationship between soil properties (fifteen variables) and tree parameters (wood density, total crown area, height, diameter and slenderness). Tree mean slenderness was positively related to the ratios (Ca + Mg):K, (Ca + Mg):(K + Na) and Ca:Mg, but not C:N (Table 6). Furthermore, slenderness positively correlated with soil P, Ca and Cu contents, but it negatively correlated with Zn content. Tree wood density positively correlated with soil organic C, Mg, Cu and Mn, but negatively correlated with Ca:Mg ratio. Whereas mean DBH positively correlated with C, P and C:N ratio, mean tree height positively correlated with Ca, P, and the ratios C:N, (Ca + Mg):K and (Ca + Mg):(K + Na), but negatively correlated with soil N content. The mean total crown area of trees was positively related to Ca:Mg ratio and soil Cu content.

**Table 6:** Correlation between selected tree parameters and soil characteristics. Correlation coefficients are shown with *p*-values in parenthesis; *p < 0.05; **p < 0.01.

	Mean WD (g cm^−3^)	Total CA (m^2^ plot^−1^)	Mean H (m)	Mean DBH (cm)	Mean SL
(Ca + Mg):K	− 0.049 (0.188)	− 0.018 (0.226)	0.202 (0.047)*	0.115 (0.115)	0.218 (0.039)*
(Ca + Mg):(K + Na)	0.018 (0.227)	0.038 (0.202)	0.268 (0.020)*	0.193 (0.052)	0.256 (0.023)*
C (Mg ha^−1^)	0.324 (0.008)**	0.051 (0.186)	0.196 (0.051)	0.216 (0.040)*	0.068 (0.165)
C:N	0.164 (0.072)	0.155 (0.079)	0.479 (< 0.001)**	0.416 (0.001)**	− 0.004 (0.245)
Ca:Mg	− 0.222 (0.037)*	0.233 (0.032)*	− 0.021 (0.223)	− 0.030 (0.212)	0.301 (0.012)*
Ca (kg ha^−1^)	0.015 (0.231)	0.000 (0.249)	0.200 (0.048)*	0.153 (0.081)	0.307 (0.011)*
Cu (kg ha^−1^)	0.204 (0.046)*	0.322 (0.008)**	0.086 (0.145)	0.135 (0.095)	0.325 (0.008)**
ECEC (cmolC kg^−1^)	0.048 (0.189)	− 0.074 (0.159)	0.104 (0.126)	0.152 (0.082)	0.194 (0.052)
K (kg ha^−1^)	0.181 (0.060)	0.058 (0.177)	− 0.028 (0.214)	− 0.001 (0.248)	0.111 (0.118)
Mg (kg ha^−1^)	0.212 (0.042)*	− 0.154 (0.080)	0.181 (0.060)	0.136 (0.0.095)	0.056 (0.179)
Mn (kg ha^−1^)	0.308 (0.011)*	0.084 (0.147)	0.114 (0.116)	0.131 (0.099)	− 0.149 (0.084)
N (kg ha^−1^)	0.115 (0.115)	0.080 (0.152)	− 0.203 (0.047)*	− 0.192 (0.053)	0.063 (0.171)
Na (kg ha^−1^)	− 0.140 (0.092)	− 0.088 (0.142)	0.073 (0.160)	− 0.009 (0.238)	0.102 (0.128)
P (kg ha^−1^)	0.163 (0.072)	0.172 (0.066)	0.243 (0.028)*	0.277 (0.017)*	0.260 (0.022)*
Zn (kg ha^−1^)	0.047 (0.191)	0.005 (0.243)	0.042 (0.196)	− 0.059 (0.176)	− 0.224 (0.036)*

*ECEC* effective cation exchange capacity, *WD* wood density, *H* tree height, *DBH* diameter at breast height, *CA* total tree crown area, *SL* slenderness.

### Biophysical factors affecting tree parameters

Tree species richness, diversity, basal area and density positively correlated with slenderness while tree species richness, diversity and basal area and standing litter were positively related to wood density (Table 7). Tree basal area correlated with mean tree height and DBH whilst tree density negatively correlated with DBH. The mean total crown area of trees was positively related to tree species richness, diversity and basal area.

**Table 7:** Correlation between selected tree parameters and stand characteristics. Correlation coefficients are shown with *p*-values in parenthesis; significant at *p = 0.05 and **p = 0.01.

	Mean WD (g cm^−3^)	Total CA (m^2^ plot^−1^)	Mean H (m)	Mean DBH (cm)	Mean SL
S (per plot)	0.208 (0.039)*	0.687 (< 0.001)**	0.065 (0.165)	− 0.023 (0.219)	0.515 (< 0.001)**
H (per plot)	0.201 (0.043)*	0.618 (< 0.001)**	0.071 (0.158)	− 0.030 (0.210)	0.492 (< 0.001)**
BA (m^2^ per plot)	0.232 (0.028)*	0.696 (< 0.001)**	0.385 (0.002)**	0.469 (< 0.001)**	0.229 (0.029)*
LS (Mg ha^−1^)	0.197 (0.045)*	0.178 (0.056)	0.136 (0.089)	0.113 (0.111)	0.129 (0.095)
TD (ha)	− 0.117 (0.107)	0.086 (0.140)	− 0.117 (0.107)	− 0.283 (0.013)*	0.359 (0.003)**

*S* tree species richness, *H* Shannon diversity, *BA* basal area, *LS* oven dry weight of standing litter, *TD* tree density, *WD* wood density, *H* tree height, *DBH* diameter at breast height, *CA* total tree crown area, *SL* slenderness.

## Discussion

### Species-specific allometries of shade trees on cocoa farms

The diameter at breast height (DBH) explained variation in crown area better than tree height (Table 3). We propose three reasons for this finding. Firstly, trees providing shade for cocoa trees are generally sparsely distributed resulting in greater lateral empty space and light availability, which promotes crown expansion and a corresponding thickening of the stem to provide mechanical support^19,37,45^. Mechanical constraints exerted by the leaves and branches, hydraulic constraints (as a consequence of the supply of water and nutrients through the stems to the leaves) and metabolic constraints (as a consequence of the metabolic demand of living cells in the stem relative to the quantity of photosynthetic sources) and their interaction with climatic, floristic and edaphic factors have been proposed by several authors [e.g., Refs.^46–48^] to explain the CA-DBH allometry. Our finding that the CA-DBH allometry conformed to the predictions of the MST supports this notion.

Secondly, it may be related to factors influencing growth in height or crown size for a given individual tree. For example, the same species can have different heights for a given diameter due to factors such as exposure to wind, slope or soil characteristics^49–51^. Given that shade trees on cocoa farms are sparsely distributed with their canopies emerging above the cocoa trees to provide shade for them, the impact of wind on these trees become more intense which may result in the thickening of the stems to prevent bending and breakage^1,19,21,37^. Furthermore, the correlation between mean DBH, mean tree height and mean tree crown area with soil nutrients and their ratios as well as stand characteristics such as tree density, basal area and species richness (Tables 6, 7) suggests their influence on tree parameters. Some studies [e.g., Refs.^21,51^] have demonstrated that tree density was the main biological variable affecting the H-DBH allometry of trees, while other studies [e.g., Refs.^52,53^] reported that as basal increases, trees become shorter. In sparse tree stands, such as our study area, larger basal area (thick stems) may provide stability against wind pressures, which explains the positive correlation between tree basal area and height contrary to the report of Zhang et al.^52^ and Lu et al.^53^ who studied dense stands where light is generally the limiting factor.

Thirdly, the fact that DBH was a better predictor of CA compared to H may be attributable to the fact that growth in tree height terminates when maximum height is attained while growth in diameter is indeterminate^11,19,21^. Since tree height terminates asymptotically at a point, it ceases to grow together with CA, possibly reducing its ability to predict CA, while DBH continues to grow together with CA, increasing its ability to predict CA. However, our results suggest growth in tree height had not terminated since the scaling exponent of the CA-H allometry was larger than both the CA-DBH and H-DBH allometries. Almost all the forest tree species evaluated in this study were pioneer trees or non-pioneer light demanders; these tree species are fast growing and site conditions such as density and available soil nutrients may amplify their growth rate.

We found that the mean scaling exponent was largest for the CA-H allometry, intermediate for the CA-DBH allometry and lowest for the H-DBH allometry (Table 3). This suggests that the stems of trees on the cocoa farms grew faster relative to their crown areas than they did relative to their height and their height grew faster together with crown area than their diameter grew together with either their height or crown area. Crown area increased with height because taller trees have larger respiration load; the larger crowns allow them to optimise the amount of photosynthesizing leaf area^34,35,54^. Crown expansion enables pioneer tree species to increase their leaf area and the number of apical meristems, occupy greater space, reduce self-shading and out-compete their neighbours through over-shading. Thus, the larger scaling exponents of the CA-H allometry than the H-DBH and the CA-DBH allometries may be due to competition for light since almost all of the evaluated shade tree species in this study were pioneer tree species^35,36,44^ or edaphic factors such as soil fertility^53^. Even though shade trees are sparsely distributed in cocoa farms, the densely planted cocoa trees which normally characterise cocoa farms may trigger height growth in shade trees, particularly, young trees. Increased tree crown size and height enables them to capture more light for growth and productivity^36,53,55^. Moreover, it is only large-statured trees that usually show asymptotic H-DBH relationships, but our data included trees with DBH ≥ 5 cm. Furthermore, in Bolivian forests, Poorter et al.^54^ reported that a quarter of tree species they evaluated did not conform to the asymptotic H-DBH allometry prediction.

The scaling exponents of tree species were significantly different, suggesting that ontogenetic patterns are highly species-specific^54,56^. The mean scaling exponent for the H-DBH allometry converges with the prediction made by the elastic and constant stress similarity theories^26,46,57,58^. Contrary to the results of Blanchard et al.^37^ which rejected the prediction of a universal scaling exponent for the H-DBH allometry by the MST, the scaling exponents of the H-DBH allometry were similar to the prediction of the MST. The mean scaling exponent of the CA-H allometry diverged from the predictions of the MST and stress similarity models, possibly due to floristic and edaphic factors [Table 4, Refs.^21,36,56^]. Furthermore, the observed variations may reflect intra-specific variability in response to stand characteristics (e.g., density) and the availability of resources^21,45,54^.

Asare and Anders (1) developed species-specific allometries between DBH and CA for recommended and commonly used shade trees in cocoa agroforestry systems in Ghana and indicated that the recommended canopy cover for cocoa agroforestry systems by the Cocoa Research Institute of Ghana (CRIG) must account for the fact that canopy cover increases with tree age and size. Our work complements their study, providing allometries for the three-dimensional structure of trees and covering shade trees not included in their study. Tiralla et al.^11^ developed and demonstrated the transferability of allometries between easily measurable tree parameters (e.g., DBH) and parameters which are more difficult to measure in dense cocoa systems (e.g., crown radius) in Indonesia. The equations provided in our study can be used to obtain information on differences in tree height and canopy cover of commonly used shade trees in cocoa farms. For example, both *Terminalia ivorensis* and *Newbouldia laevis* are recommended for use as shade trees in cocoa farms, but on the average, one *T. ivorensis* tree (mean CA = 91 m^2^) can provide the shade cover provided by five individuals of *N. laevis* (mean CA = 17 m^2^) [see Refs.^1,11^]. That notwithstanding, the quantity and quality of light transmitted by shade trees will differ on the basis of crown size and depth. In addition, relative canopy projection area (RSCA), a proxy for both leaf area and light interception and an indication of shade intensity^18^, increased with shade tree species richness and diversity and shade tree density (Fig. 3). Given that shade tree density and species richness were better predictors of RSCA, manipulating tree density may lead to optimum shade intensity and minimise multi-coverage without compromising shade tree species richness and diversity^2^. For example, Bagin et al.^59^ reported that plots with high shade tree diversity with variable stem density and sizes resulted in a varied transmission of light ranging from 9.4 to 80.1%.

Furthermore, under the REDD + (Reduced Emissions from Deforestation and Forest Degradation), Ghana defines a forest as a minimum of 1 ha with trees taller than 5 m having a minimum canopy cover of 15%^32^. The equations we provide in this study are useful in selecting shade trees and monitoring their growth in cocoa agroforestry systems which is a key step to addressing REDD + challenges such as optimising shade tree density on cocoa farms. Species-specific allometric equations take into account the unique growth patterns and structural characteristics of individual tree species^1,11,15^. Therefore, our equations may potentially improve the estimation of biomass, carbon sequestration, and ecosystem services in agroforestry systems in relation to REDD+, even though this was not directly evaluated in the study. Thus, the assessment of the impact of species-specific structural allometries on biomass estimation in agroforestry systems are urgently needed. Moreover, larger sample size and diameter ranges may also provide additional benefits in relation to their range of applicability. That notwithstanding, the availability of species-specific allometries is a key step to linking field data with remotely sensed data for broad scale estimation of stand parameters such as the canopy cover of shade trees on cocoa farms. To overcome the challenge of limited data on difficult-to-measure tree attributes, Jucker et al.^43^, developed a global tree allometry and crown architecture database (Tallo database) for 498,838 individual trees. This is a huge step forward, however, 64% of the species evaluated in this study are not captured in the Tallo database.

### Stand and soil factors influencing tree slenderness

We found that tree slenderness coefficients, an index indicating the resistance of trees to windthrow, were positively related to tree density, species richness and diversity, and tree basal area; our results are similar to Wang et al.^29^ who reported that the slenderness coefficients of boreal mixed wood forests were positively correlated with stand parameters. Increased species richness and diversity leads to increased competition of underground roots for soil resources and aboveground parts for light resources, which results in variations in tree growth and slenderness^60,61^. Denser stands are usually associated with lower DBH growth and this may explain why slenderness was positively related to tree density. Our mean slenderness coefficients (per plot) range from 46.7 to 184.0 with 10% of the studied cocoa farms having slenderness coefficient ≥ 100, thus, are potentially at high-risk of windthrow^29,62^. Higher slenderness coefficients indicate shorter crowns, higher centre of gravity, and a poorly developed root system; making such trees highly susceptible to wind damage.

The mean slenderness coefficients of the species we evaluated ranged from 38.8 (*Mangifera indica*) to 102.0 (*Holarrhena floribunda*). Three of the nine forest tree species had slenderness coefficients in the range 80.0–102.0 (Table 5) and this indicates their susceptibility to wind damage^62–64^. Both of the fruit trees demonstrated greater resistance to wind damage compared to the forest trees (Tables 2, 5). The lower slenderness coefficients for fruit trees versus higher slenderness coefficients for forest trees may be related to differences in resource allocation. While fruit trees may exhibit larger crowns and invest in fruit and seed production, the forest trees, which were all fast-growing species, are likely to invest in height and crown growth to maximise light capture. The consequence of the differences in resource allocation are shorter and thicker stems for the fruit trees, making them more stable, whereas the fast-growing species would be taller and their stem will be just strong enough to bear the weight of their crowns and height, possibly making them more susceptible to wind damage^55,62^.

Slenderness coefficients decreased with increasing crown area (Fig. 2) possibly because larger crown sizes trigger the thickening of tree stems to provide mechanical support, thus increasing their resistance to wind damage^29,55^. Similarly, Giagli et al.^62^ reported that the slenderness coefficient of silver birch was negatively related to its crown area. The lack of fit between slenderness coefficients and CA for the species *M. zechiana, S. campanulata* and *N. laevis* may be due to confounding factors or lack of power which calls for investigating these species at a broader scale. At the plot level, our data show that tree slenderness coefficients correlated with critical soil nutrients and their ratios (Table 6). Generally, better site conditions result in higher tree slenderness coefficients due to fast growth ^29^.

### Biophysical factors affecting tree parameters

Basal area (BA), which can be considered as a proxy for competition index, correlated with mean tree height and diameter suggesting that increase in BA is associated with increase in mean DBH and H. Variation in the DBH-H allometry is strongly influenced by BA^19,21^. Zhang et al.^52^ and Lu et al.^53^ reported that trees tended to be taller with lower BA (lower competition); our results are contrary to their finding possibly because of differences in context or due to the fact that the species we studied were all pioneers or non-pioneer light demanders, which grow rapidly to reach the canopy level. From literature, wood density is affected by several factors such as tree age, species and size, growth rate, and their interaction^65–68^. Therefore, our finding that stand characteristics such as tree species richness, diversity and basal area and standing litter were positively related to wood density is consistent with existing literature.

### Soil factors affecting tree parameters

Soil fertility accelerates the growth of H^53,61^. The mean tree height at the plot level correlated with soil P and Ca contents and the ratios C:N, (Ca + Mg):K and (Ca + Mg):(K + Na), suggesting the influence of edaphic factors. Specifically, Chen et al.^61^ reported that soil organic carbon and available soil P and N stimulate increase in biomass, DBH and H. Soils of cocoa systems in the study area are generally acidic, Ca^2+^ counterbalances the toxicity of aluminium ions and maintains the homeostasis of intracellular ions, thus, enhancing the survival and growth of trees^33,61^. Moreover, soil P promotes efficient use of soil nutrients by affecting plant root structure and promoting the formation and growth of fine roots, lateral roots and secretion of root exudates^60^. Similar to our results, Perumal et al.^69^ reported that soil available P correlated with tree H and DBH.

Our results show that wood density was positively correlated with soil parameters such soil organic C, Mg, Cu and Mn, but negatively related to Ca:Mg ratio. These results are indicative of the influence of soil parameters on tree wood density^70,71^. For example, the accumulation of soil organic carbon promotes the availability of other soil nutrients which influences plant growth and development, including wood density^61^.

## Conclusion

The study describes the structure of associated trees on cocoa farms, providing species-specific allometries which are a step forward in linking field data with remotely sensed data for broad scale estimation of the biomass of commonly used shade trees on cocoa farms. DBH remains a better predictor of crown form than H, thus, difficult-to-measure tree parameters may be estimated from easy-to-measure ones such as DBH. The scaling exponents of the H-DBH allometry were narrower and smaller compared to the CA-DBH and CA-H allometries. All tree allometries, i.e., H-DBH, CA-DBH and CA-H, conformed to the metabolic scaling theory. There is an inverse relationship between slenderness coefficients and tree crown area. Soil and stand characteristics influence the structural characteristics of shade trees which affects tree allometries. Relative canopy projection area, a proxy for shade intensity, was better related to tree density and richness than tree diversity, suggesting that management of individual species’ stem density may lead to desired shade intensity, but not at expense of shade tree richness and diversity. It is urgent to compare allometries of shade trees in agroforestry systems to their counterparts in forests to provide complementary information on their structural development. It is also critical to link field data on species-specific allometries with remotely sensed data for broadscale analysis of cocoa agroforestry systems.

### Supplementary Information

Supplementary Information.

## Data Availability

The manuscript and its appendices contain all the relevant data. All datasets generated and/or analysed during the current study are available from the corresponding author on reasonable request.
