# Supplementary material for: Allometric relationships between stem diameter, height and crown area of associated trees of cocoa agroforests of Ghana
Source: Sci Rep. 2023 Sep 9;13:14897. doi: 10.1038/s41598-023-42219-6 (PMC10492788; doi:10.1038/s41598-023-42219-6)
Supplement: Supplementary file 1 — Supplementary Information. [file 41598_2023_42219_MOESM1_ESM.pdf]

Allometric relationships between stem diameter, height and crown area of associated trees of cocoa agroforests of Ghana

Michael Asigbaase[1*], Evans Dawoe[2], Simon Abugre[1], Boateng Kyereh[3] and Collins Nsor Ayine[4]

Supplementary Information 1: Soil data collection, processing and chemical analysis methods

The soil samples were collected from two depths, namely 0-15cm and 15-30cm, for the purpose of conducting a chemical analysis and soil bulk density determination. A 5cm-bladed Eijkelkamp soil auger was utilized to extract five soil samples from each depth in each plot. The auger was marked at the corresponding depths, and manually driven into the soil to extract the samples. The collected samples from each layer in each plot were combined, thoroughly mixed, and subsampled for further analysis. The soil subsamples were dried in an oven at 105°C for 48 hours, sieved through a 2mm mesh, and ground in an agate ball mill at 290rpm for 15 minutes prior to chemical analysis. To determine soil bulk density, two soil samples per depth per plot were collected using 139cm^3 bulk density cylinders. These samples were obtained from the soil wall after digging. All samples were collected on the same day to ensure consistency.

Soil water content (%WC) was determined using the formula WC= $[(M_1 – M_2)/M_2] \times 100$, where M_1 represents the fresh weight of soil and M_2 is the weight of soil that was oven-dried at 105°C for 48 hours. Soil bulk density was estimated as BD (g cm^{-3}) = $[(W_1 – W_2)/V] \times (100 - \%CF)/100$, where BD represents soil bulk density, W_1 and W_2 are the weights of empty trays and oven-dried soils in trays, respectively, and V represents the volume of the bulk density cylinder. The coarse soil fraction (CF) was used to adjust for the effect of stone

fragments on bulk density. Soil particle size distribution was determined using laser ablation (Bechman Coulter LS 200) and classified into textural classes based on the USDA soil triangle (Soil Survey Division Staff, 1993).

Soil pH was measured in a 1:2.5 soil:solution slurry using a pH meter (pH 209, Hanna Instruments), which was calibrated with pH 4.01 and 7.00 buffer solutions. Electrical conductivity (EC, mS cm^{-1}) was also measured in the same soil slurry using a portable electrical conductivity meter (Combo pH and EC, Hanna Instruments), which was calibrated with 1413 μS cm^{-1} standard solution. To determine Olsen extractable P (hereafter P), soil samples (2 g) were extracted with 30 ml of 0.5 M sodium bicarbonate, which was thoroughly mixed with 0.05% w/v polyacrylamide, shaken, and centrifuged at 3500 rpm for 15 min. Phosphorus in the extract was estimated at 880 nm in a 1 cm cell using a spectrophotometer and the blue phospho-molybdate method with ascorbic acid as a reducing agent.

Total soil organic carbon (SOC) and total nitrogen (N) were analyzed using a CN analyzer [Thermo Scientific™ Flash™ 2000 Organic Elemental Analyzer (OEA)]. Extractable Ca, Mg, K, and Na, as well as the concentrations of Mn, Al, Cu, and Zn, were determined using ICP-MS (Thermo Scientific™ iCAP™ TQ) after extracting 2 g of each soil sample with 20 ml of 1 M NH_4NO_3, centrifuging at 3500 rpm for 30 min, filtering, and diluting 1 ml of the supernatant with 9 ml of 2% HNO_3. Effective cation exchange capacity (ECEC) was determined as the sum of exchangeable bases and exchangeable acidity. Nutrient stocks (Mg or kg ha^{-1}) were estimated as the product of nutrient concentration, bulk density, depth, and unit conversion factor. The total nutrient stocks for the 0-30 cm depth were used for the correlation analysis involving soil nutrients.

Supplementary Tables

Supplementary Table 1: Structural attributes of cocoa agroforestry systems at Suhum.

**Variable**	**Mean**	**SEM**	**Min**	**Q1**	**Median**	**Q3**	**Max**
Cocoa age (years)	24.85	1.64	10.00	15.00	22.00	35.00	50.00
Farm size (ha)	1.57	0.27	0.24	0.63	1.00	2.00	10.12
Strata	1.75	0.11	0.00	1.00	2.00	2.00	3.00
Tree density (*per* ha)	113	15.30	0	48	96	144	672
Cocoa density (*per* ha)	1155	42.40	304	992	1120	1388	1744
Fruit plant density (*per* ha)	207	29.80	0.00	64	168	284	832
Stand basal area (m^2 ha^{-1})	9.85	0.85	0.00	4.57	10.47	13.97	23.52
Cocoa basal area (m^2 ha^{-1})	10.72	0.53	1.42	9.01	10.70	12.54	23.21
Spp. richness (S, *per* plot)	4.33	0.38	0.00	2.25	4.00	6.00	13.00
Shanon diversity (H, *per* plot)	1.23	0.09	0.00	0.77	1.33	1.72	2.27
SLD (Mg ha^{-1})	3.87	0.06	2.82	3.51	3.89	4.19	4.76
Mean slenderness	71.74	4.23	0.00	59.28	72.21	83.73	184.00
Mean H (m)	14.91	0.77	0.00	12.22	15.12	18.23	24.63
Mean DBH (cm)	24.43	1.55	0.00	17.47	22.81	31.00	47.98
Mean wood density (g cm^{-3})	1.38	0.85	0.00	0.49	0.55	0.62	41.25
Total CA (m^2)	291.50	29.70	0.00	116.70	247.10	411.80	841.70
RSCA (%)	46.64	4.75	0.00	18.68	39.53	65.89	134.67

SLD is oven dry standing litter density, H is height (m), DBH is diameter at breast height (cm), CA is canopy area, and RSCA is relative sum of crown projection area.

Supplementary Table 2: Soil attributes of cocoa agroforestry systems at Suhum.

Soil attribute	Mean	SEM	Min	Q1	Median	Q3	Max
Effective CEC (cmolC kg^{-1})	11.23	0.73	5.04	0.74	7.09	10.60	14.62
Ca:Mg	7.60	0.42	2.88	4.15	5.89	7.09	8.25
(Ca+Mg):K	25.63	1.71	11.82	6.82	18.54	23.97	31.76
(Ca+Mg):(K+Na)	22.09	1.38	9.53	6.46	16.58	20.48	27.16
C:N	9.34	0.57	3.81	1.58	7.18	9.88	11.14
C (Mg ha^{-1})	54.70	2.45	16.98	20.38	43.70	54.52	62.43
N (kg ha^{-1})	6.66	0.51	3.55	1.03	4.42	5.78	8.47
P (kg ha^{-1})	39.58	2.92	20.25	6.24	22.02	39.46	53.99
K (kg ha^{-1})	295.80	25.00	173.30	103.50	179.80	247.10	374.10
Na (kg ha^{-1})	37.56	3.39	23.46	12.91	21.81	30.88	44.78
Ca (kg ha^{-1})	5705.00	392.00	2717.00	2194.00	3662.00	5342.00	6880.00
Mg (kg ha^{-1})	776.70	47.00	325.30	287.70	524.50	742.00	999.30
Mn (kg ha^{-1})	238.00	24.10	166.90	32.10	110.70	210.20	304.80
Cu (kg ha^{-1})	0.28	0.04	0.27	0.00	0.03	0.20	0.49
Zn (kg ha^{-1})	1.22	0.21	1.47	0.00	0.25	0.79	1.51

CEC is cation exchange capacity, SEM is standard error of mean, Q1 is first quantile, and Q3 is third quantile.
